# European Regional and Local Health Authorities (EUREGHA)

**DOI:** 10.1093/eurpub/ckac129.489

**Published:** 2022-10-25

**Authors:** M Calabro

**Affiliations:** European Regional and Local Health Authorities, Brussels, Belgium

## Abstract

EUREGHA's vision is to ensure that the local and regional perspective is represented in EU health policy because local and regional authorities are the natural interface between citizens and European institutions, being the bridging bodies between policies and practices and the closest organizations to the concept of communities. We are the only European network prioritizing the representation of local and regional health authorities at EU level, as they fulfil a key role in improving efficiency, quality, and accessibility of healthcare systems and services. EUREGHA understands their specific needs and works tirelessly to represent regional and local health authorities in EU health policy, to amplify their voices as a means to improve European public health and healthcare. Through advocacy, policy monitoring, profile promotion, partnerships, and project development, EUREGHA facilitates and promotes collaboration between its members, EU institutions, pan-European health networks and other healthcare stakeholders. Network activities and projects are implemented upon a strategic background of “ways to make things happen” such as value based healthcare, smart specialization strategies and skills for innovation and digital transformation - on one side - and along thematic and field objectives such as cross-border healthcare, cancer, obesity, mental health and ageing - on the other side. Lower Austria is a long-standing member and Vice-Chair of EUREGHA.

